# Gross hematuria after SARS-CoV-2 vaccination: questionnaire survey in Japan

**DOI:** 10.1007/s10157-021-02157-x

**Published:** 2021-11-13

**Authors:** Keiichi Matsuzaki, Ryousuke Aoki, Yoshihito Nihei, Hitoshi Suzuki, Masao Kihara, Takashi Yokoo, Naoki Kashihara, Ichiei Narita, Yusuke Suzuki

**Affiliations:** 1 Joint Research Team From Japanese Society of Nephrology and the Progressive Renal Diseases Research, Research on Intractable Disease, from the Ministry of Health, Labour and Welfare of Japan, Special Study Group for IgA Nephropathy, Tokyo, Japan; 2 Kyoto University Health Service, Kyoto, Japan; 3 Department of Nephrology, Juntendo University Faculty of Medicine, Hongo 2-1-1, Bunkyo-ku, Tokyo, 113-8421 Japan; 4 Division of Kidney and Hypertension, Department of Internal Medicine, Jikei University School of Medicine, Tokyo, Japan; 5 Department of Nephrology and Hypertension, Kawsaki Medical School, Kurashiki, Japan; 6 Division of Clinical Nephrology and Rheumatology, Niigata University Graduate School of Medical and Dental Sciences, Niigata, Japan; 7 Division of Nephrology, Department of Internal Medicine, Faculty of Medicine, Juntendo University, 2-1-1 Hongo, Bunkyo-ku, Tokyo, 113-8421 Japan

**Keywords:** SARS-CoV-2 vaccination, Coronavirus 2019, Gross hematuria, IgA nephropathy, mRNA vaccination

## Abstract

**Background:**

Recent clinical reports indicate a correlation between gross hematuria after the coronavirus 2019 (COVID-19) vaccination in patients with glomerulonephritis, especially immunoglobulin A nephropathy (IgAN). Furthermore, healthcare workers in Japan were initially vaccinated with an mRNA vaccine from February 17, 2021, and some of them experienced gross hematuria after receiving the vaccination.

**Methods:**

We conducted a web-based survey of the councilor members of the Japanese Society of Nephrology (581 members, 382 facilities) to elucidate the relationship between gross hematuria and COVID-19 vaccination.

**Results:**

In the first survey, 27 cases (female: 22, 81.5%) of gross hematuria were reported after receiving a COVID-19 vaccination. Of them, 19 (70.4%) patients were already diagnosed with IgAN at the occurrence of gross hematuria. Proteinuria appeared in eight of the 14 (57.1%) cases with no proteinuria before vaccination and hematuria in five of the seven (71.4%) cases with no hematuria before vaccination. The second survey revealed that a renal biopsy was performed after vaccination in four cases, all of whom were diagnosed with IgAN. Only one case showed a slightly increased serum creatinine level, and no patients progressed to severe renal dysfunction.

**Conclusion:**

This study clarified the clinical features of gross hematuria after a COVID-19 vaccination. Because there was no obvious progression to severe renal dysfunction, safety of the COVID-19 vaccination is warranted at least in the protocol of inoculation twice.

**Supplementary Information:**

The online version contains supplementary material available at 10.1007/s10157-021-02157-x.

## Introduction

The effective control of coronavirus disease 2019 (COVID-19) can only be achieved by implementing a global vaccination strategy. Recently, several types of vaccines against severe acute respiratory syndrome coronavirus 2 (SARS-CoV-2) have been developed, mainly in Western countries, and vaccination rollout has commenced [1–6]. From February 17, 2021, healthcare workers in Japan were initially vaccinated with an mRNA vaccine (BNT162b2 [COMIRNATY], Pfizer-BioNTech; Pfizer, New York, NY, and BioNTech, Mainz, Germany) [7]. To date, a total of approximately one hundred and sixty million vaccines have been administrated [8].

Several studies have reported the appearance of gross hematuria after COVID-19 vaccination in patients with glomerulonephritis, especially those with immunoglobulin A nephropathy (IgAN) [9–13]. A recent systematic review reported a higher prevalence of patients with IgAN in Asian than in Caucasian populations [14]. Thus, investigation of the frequency and clinical features of gross hematuria after receiving the COVID-19 vaccination in Japan and Asia is vital for the clinical management of IgAN under the current pandemic situation. To this end, the joint research team from the Japanese Society of Nephrology and the Progressive Renal Diseases Research, Research on intractable disease, from the Ministry of Health, Labour and Welfare of Japan conducted a clinical survey of gross hematuria associated with COVID-19 vaccination using a web-based questionnaire.

## Methods

The first survey comprised a web-based questionnaire that was emailed to councilor members of the Japanese Society of Nephrology (581 members in 382 facilities) between June 2 and June 20, 2021. The questionnaire asked about cases of gross hematuria that were observed after receiving the COVID-19 vaccination and their outcomes (Table 1). Then, between June 9 and June 19, 2021, a second survey was emailed to the members who reported cases of gross hematuria. The second survey asked about the incidence of elevated serum creatinine levels, the status of proteinuria and hematuria, and pathological diagnosis (if a renal biopsy was performed).

**Table 1:** Contents of questionnaire in first survey

Question number	Questions	Response
Q1-1	How old is this patient?	1. ≤ 19
		2. 20–29
		3. 30–39
		4. 40–49
		5. 50–59
		6. 60–69
		7. ≥ 70
Q1-2	What is the patient's gender?	1. Male
		2. Female
Q1-3	Has this patient undergone a renal biopsy? If yes, what was their diagnosis?	1. Diagnosed with IgA nephropathy
		2. Did not perform a renal biopsy
Q1-4	Check all the following treatments used in this case	1. Tonsillectomy
		2. Steroid pulse therapy
		3. Oral corticosteroid
		4. RAS-I
		5. Antiplatelet drugs
		6. Others
Q2-1	What type of vaccine was used in this patient?	1. COMIRNATY Intramuscular Injection (Pfizer-BioNTech)
		2. COVID-19 Vaccine Moderna Intramuscular Injection (Moderna/Takeda)
		3. VAXZEVRIA Intramuscular Injection (AstraZeneca)
		4. Others
Q2-2	After what vaccination did you point out the gross hematuria?	1. After first dose vaccination
		2. After second dose vaccination
		3. Both first dose and second dose vaccination
		4. Others
Q2-3	How many days after vaccination did the gross hematuria appear?	1. ≤ 1 day
		2. 2−3 days
		3. 4−7 days (almost 1 week)
		4. 8−14 days (almost 2 weeks)
		5. 15− 28 days (almost 3–4 weeks)
		6. Others
Q2-4	How long did the gross hematuria continue?	1. ≤ 1 day
		2. 2−3 days
		3. 4− 7 days (almost 1 week)
		4. Over 8 days
		5. Others
Q2-5	Did an adverse reaction to the vaccination occur in the patient with the gross hematuria?	1. Did not experience an adverse reaction
		2. Unknown
		3. Fever (≥ 37.5 ℃)
		4. Headache
		5. General fatigue
		6. Chills
		7. Muscle pain
		8. Joint pain
		9. Others
Q3-1	Did this patient have proteinuria prior to the vaccination?	1. Yes
		2. No
Q3-2	Was there an appearance or worsening of proteinuria after the disappearance of gross hematuria?	1. Yes
		2. No
		3. Others
Q3-3	Did this patient have hematuria prior to the vaccination?	1. Yes
		2. No
Q3-4	Was there an appearance or worsening of hematuria after the disappearance of gross hematuria?	1. Yes
		2. No
		3. Others
Q3-5	Was there a worsening of renal function after disappearance of gross hematuria ?	1. Yes
		2. No
		3. Others

*COVID-19* coronavirus disease 2019; *IgA* immunoglobulin A; *RAS-I* renin–angiotensin system inhibitor

## Results

**Fig. 1:**
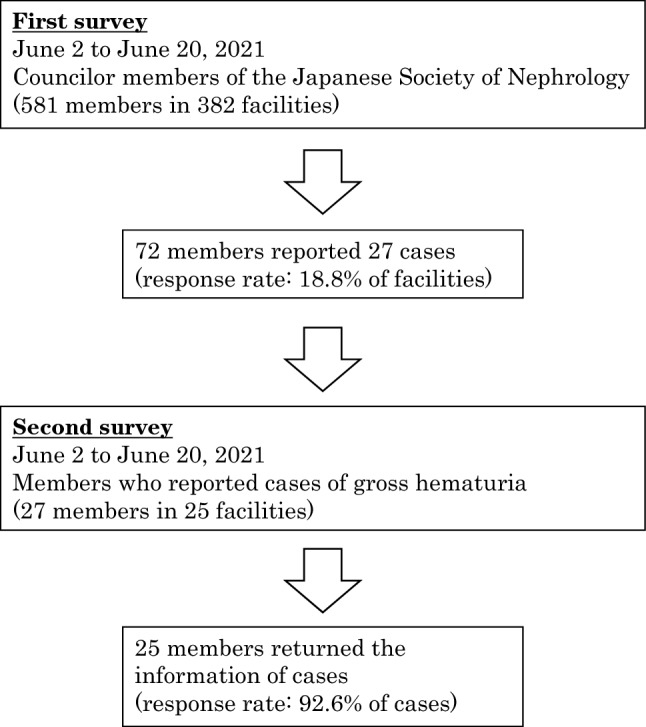
Outline of this study

**Fig. 2:**
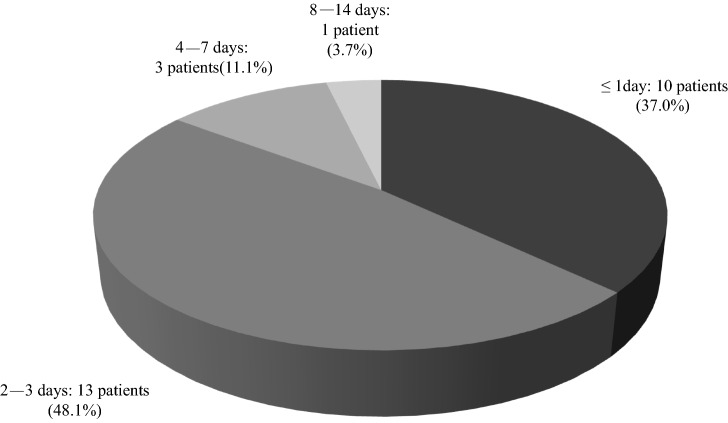
Length of time between COVID-19 vaccination and the appearance of gross hematuria

**Fig. 3:**
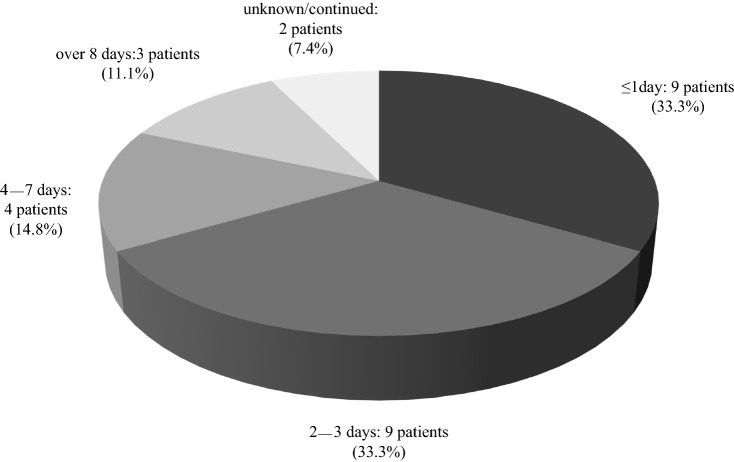
Duration of gross hematuria after receiving the COVID-19 vaccination

Figure 1 shows the outline of this study. In the first survey, 72 members (response rate: 18.8% of facilities) reported 27 cases of gross hematuria after receiving the COVID-19 vaccination. The baseline characteristics of the patients with gross hematuria are presented in Table 2. Most of the patients were aged 20–29 years (40.7%), and those aged 20–39 years accounted for 66.7% of the study cohort. Female patients comprised 81.5% of cases. Furthermore, 88.9% of cases occurred after vaccination with BNT162b2 (Pfizer-BioNTech). Of the 19 cases (70.4%) already diagnosed as IgAN, eight cases (29.6%) did not undergo renal biopsy for diagnosis. Figure 2 shows the length of time between COVID-19 vaccination and the appearance of gross hematuria, with 23 cases (85.2%) occurring within 3 days after the vaccination. Figure 3 shows the duration of the gross hematuria, with 18 cases (66.7%) disappeared gross hematuria until 3 days after its appearance. Tables 3 and 4 show the details of urinary abnormalities after receiving the COVID-19 vaccination. Proteinuria appeared in eight of the 14 (57.1%) cases with no proteinuria before vaccination. Hematuria appeared in five of the seven (71.4%) cases with no hematuria before vaccination. All the four patients who received tonsillectomy did not show the worsening proteinuria and hematuria.

**Table 2:** Baseline characteristics of the 27 cases with the appearance of gross hematuria after receiving a COVID-19 vaccination

Characteristic	Cases (*n* = 27)
Age	
20–29	11
30–39	7
40–49	5
50–59	1
60–69	1
≥ 70	2
Sex	
Male	5
Female	22
Treatments (multiple answers allowed)	
Tonsillectomy	4
Steroid pulse therapy	3
Oral corticosteroid	3
RAS-I	4
Antiplatelet drugs	2
Others	1
Never	5
Did not answer	11
Type of the vaccine	
COMIRNATY Intramuscular Injection (Pfizer-BioNTech)	24
COVID-19 Vaccine Moderna Intramuscular Injection (Moderna/Takeda)	2
Unknown	1
Vaccination dose	
First dose	8
Second dose	17
Both first and second doses	2
Adverse reactions (multiple answers allowed)	
Fever (≥ 37.5 ℃)	17
Fatigue	9
Headache	4
Chills	1
Muscle pain	1
Pain at the application site	1
Back pain	1
None	3
Unknown	5

*COVID-19* coronavirus disease 2019; *RAS-I* renin–angiotensin system inhibitor

**Table 3:** Details of proteinuria after receiving a COVID-19 vaccination

Details of proteinuria	All cases (*n* = 27)	Cases diagnosed IgA nephropahty (*n* = 19)	Cases without diagnose (*n* = 8)
Cases with proteinuria before the vaccination			
Exacerbated proteinuria	4	3	1
Did not exacerbated proteinuria	8	6	2
Unknown	1	1	0
Cases with no proteinuria before the vaccination			
Appearance of proteinuria	8	5	3
No proteinuria	5	4	1
Unknown	1	0	1

**Table 4:** Details of hematuria after receiving a COVID-19 vaccination

Details of hematuria	All cases (*n* = 27)	Cases diagnosed IgA nephropahty (*n* = 19)	Cases without diagnose (*n* = 8)
Cases with hematuria before the vaccination			
Exacerbated hematuria	9	5	4
Did not exacerbate hematuria	7	6	1
Unknown	4	3	1
Cases with no hematuria before the vaccination			
Appearance of hematuria	5	3	0
No hematuria	2	2	2

In the secondary survey, information on 25 of the 27 cases was returned (response rate: 92.6%). Increased levels of urinary protein were noted in two cases (7.4%). Regarding hematuria, 23 cases (85.2%) improved as well as before vaccination. A renal biopsy was performed after vaccination in four cases, all of which were diagnosed as IgAN. Notably, only one case showed a slight increase in the level of serum creatinine, and no patients progressed to severe renal dysfunction.

## Discussion

We investigated the clinical characteristics of gross hematuria after COVID-19 vaccination in Japan. Although cases of gross hematuria after the COVID-19 vaccination were observed, only one case showed increased levels of serum creatinine. To the best of our knowledge, this is the first case series of gross hematuria after receiving the COVID-19 vaccination.

Our survey showed that gross hematuria after receiving the COVID-19 vaccination was skewed toward females, comprising 81.4% of cases. The amount of female healthcare workers (medical doctors, public health nurses, midwives, registered nurses, and licensed practical nurses) in Japan is four times that of male healthcare workers, suggesting that such gender bias might partly reflect the female-dominant occurrence of gross hematuria. Another reason is difference in immune responses between males and females. It is widely accepted that both innate and adaptive immune responses differ between males and females and likely contribute to differences between the sexes in response to vaccines [15, 16]. Therefore, the occurrence of gross hematuria may be biased toward females.

There are several reports of gross hematuria after receiving the COVID-19 vaccination [9–13]. To date, vaccinations based on several mechanisms that trigger an immune response have been administered; however, gross hematuria was only reported after receiving an mRNA vaccination. The BNT162b2 (Pfizer-BioNTech) and the Moderna mRNA-1273 (Cambridge, MA) vaccines use a nucleoside-modified, purified mRNA lipid nanoparticle-encapsulated platform. This novel RNA platform induces stronger antigen-specific cluster of differentiation (CD)4+ and CD8+ T cell responses in experimental animals [17]. Because the CD4+ and CD8+ T cells activated by vaccination produce several proinflammatory cytokines, including interferon-γ and tumor necrosis factor-α, we wondered whether these vaccines could activate or exacerbate immune-mediated glomerular disease [9] or induce de novo glomerulonephritis, particularly IgAN. In this study, three cases were newly diagnosed as IgAN by a kidney biopsy that was taken because of the appearance of gross hematuria after receiving a COVID-19 vaccination. This suggests that such immune activation may be largely related to the mechanism of onset of glomerular nephritis.

Another point of interest is the association between IgAN pathogenesis and Toll-like receptors (TLRs), which are a family of innate immune receptors whose activation is crucial to induct the innate and adaptive immune responses [18]. Increased amounts of aberrantly glycosylated IgA have been thought to the first hit in pathogenesis of IgAN [19]. We previously demonstrated the association with TLR9, which recognizes single-stranded DNA containing unmethylated CpG motifs, and the synthesis of these IgA [20, 21]. On the other hand, Zheng et al. recently showed that TLR7, which recognizes endogenous or exogenous single-stranded RNAs [22], is also involved in the production of aberrantly glycosylated IgA1 [20], indicating that there might be some link between TLR signaling and the pathogenesis of IgAN. Thus, it is possible that mRNA vaccination affects the production of aberrant glycosylated IgA via TLR signaling.

Our study had several limitations. First, there was a possibility of selection bias, because the response rate was only 18.8% from 382 facilities. Furthermore, because councilor members tended to be affiliated with the large hospitals, our results did not include patients who were followed-up in small clinics. Second, because the questionnaire was a once-off survey, the clinical course of these cases could differ depending on the timing of the response. Therefore, we plan to conduct a prospective cohort study in the future that will overcome these limitations. Third, the study population was mainly healthcare workers. Generally, healthcare workers have easy access to a hospital. This may have resulted in a high rate of diagnosis of IgAN.

In conclusion, this small survey clarified the clinical features of gross hematuria after the COVID-19 vaccination in Japan. Although the nephrologists should, therefore, carefully and periodically follow-up on the urinary findings, the safety of the COVID-19 vaccination is warranted at least in the protocol of inoculation twice because these cases showed no obvious progression to severe renal dysfunction. Further studies are necessary to investigate the underlying mechanism of gross hematuria following COVID-19 vaccination.

## Supplementary Information

Below is the link to the electronic supplementary material.

Supplementary file1 (DOCX 17 KB)
