# Supplementary material for: Gross hematuria after SARS-CoV-2 vaccination: questionnaire survey in Japan
Source: Clin Exp Nephrol. 2021 Nov 13;26(4):316–22. doi: 10.1007/s10157-021-02157-x (PMC8590432; doi:10.1007/s10157-021-02157-x)
Supplement: Supplementary file 1 — Supplementary file1 (DOCX 17 KB) [file 10157_2021_2157_MOESM1_ESM.docx]

**Supplement table 1:** Histopathological findings using the Oxford classification and Japanese histological grade which were diagnosed as IgA nephropathy after vaccination.

| Patient | Oxford Classification (MEST-C) | | | | | Japanese histological grade |
| --- | --- | --- | --- | --- | --- | --- |
|  | Mesangial proliferation (M) | Endocapillary proliferation (E) | Segmental sclerosis (S) | Tubular Atrophy and Fibrosis (T) | Crescents (C) |  |
| 1 | 0 | 0 | 0 | 0 | 0 | ⅠA/C |
| 2 | 1 | 0 | 1 | 0 | 1 | ⅠA/C |
| 3 | 1 | 0 | 1 | 0 | 0 | ⅠC |
| 4 | 0 | 1 | 0 | 0 | 1 | Ⅱ A/C |
